# High Tim-3 expression on AML blasts could enhance chemotherapy sensitivity

**DOI:** 10.18632/oncotarget.22141

**Published:** 2017-10-27

**Authors:** Liangjing Xu, Jinge Xu, Shoubao Ma, Xiaoli Li, Mingqing Zhu, Suning Chen, Yue Han, Xiaowen Tang, Zhengzheng Fu, Huiying Qiu, Jianhua Yu, Depei Wu, Xiaojin Wu

**Affiliations:** ^1^ Jiangsu Institute of Hematology, The First Affiliated Hospital of Soochow University, Suzhou, China; ^2^ Institute of Blood and Marrow Transplantation, Suzhou, China; ^3^ Collaborative Innovation Center of Hematology, Soochow University, Suzhou, China; ^4^ Key Laboratory of Thrombosis and Hemostasis of Ministry of Health, Suzhou, China; ^5^ The Second Affiliated Hospital of Xuzhou Medical University, Xuzhou, China; ^6^ The Ohio State University, Comprehensive Cancer Center, Columbus, OH, USA

**Keywords:** Tim-3, acute leukemia, expression, chemotherapy

## Abstract

T-cell immunoglobulin and mucin domain-containing molecule3 (Tim-3) represents a novel mechanism of T-cell dysfunction and exhaustion. Tim-3 has also been identified in various solid tumors. However, the role of Tim-3 expression on blast cells in acute myeloid leukemia (AML) is not well understood. In this study, we aimed to explore the role of Tim-3 in patients with de novo AML, and the correlation between Tim-3 and clinicopathological prognosis. The study cohort consisted of 76 patients with de novo non-M3 AML. These patients’ bone marrow samples were collected and then bone marrow mononuclear cells (BMCs) were isolated for flow cytometry to detect Tim-3 expression on blasts. According to FAB type, 76 diagnosed AML patients included in this study were: M0 (n=2), M1 (n=16), M2 (n=20), M4 (n=20), M5 (n=16), and M6 (n=2). A positive expression (>20%) of Tim-3 was found in 87% (66/76) of patients with AML. The average percentage of Tim-3(+) blasts in these AML patients was 58.26 ± 29.23%. Moreover, the frequency of Tim-3 high expression was higher in M4 patients than that in other AML patients according to FAB type (P=0.004). Tim-3 high expression was also closely associated with inv(16) (P=0.01) and C/EBPA mutation (P=0.03). The mutations of the following six genes, i.e., FLT3-ITD, NPM1, C-KIT, IDH1/IDH2, DNMT3A, were independent of the Tim-3 expression. Additionally, it is more likely to find higher levels of Tim-3 in the low-risk group than in the intermediate- and high-risk groups (P=0.02). The expression of Tim-3 was positively correlated with CD13 (r=0.36, P=0.001), CD34 (r=0.41, P=0.000), and CD7 (r=0.27, P=0.02) in AML patients. AML patients with high Tim-3 expression achieved significantly high complete remission (CR) rate (P=0.01), while their Tim-3 expression significantly decreased after CR (P=0.01). Blockade of Tim-3 expression on AML blasts significantly reduced the Idarubicin (IDA)-mediated suppression of cell growth and reduction of cell apoptosis *in vitro*. Collectively, our study suggests that high Tim-3 expression on AML blasts could enhances chemotherapy sensitivity.

## INTRODUCTION

T-cell immunoglobulin and mucin domain-containing molecule 3 (Tim-3) is a type I membrane protein, which is expressed on Th1 cells, dendritic cells, monocytes and CD8+T cells, and other lymphocyte subsets, mediating immune suppression through different mechanisms [1, 2]. Tim-3 has emerged as a promising target for cancer immunotherapy. Recent studies have focused on the role of Tim-3 expression on CD4+ and CD8+T cells in peripheral blood as well as within tumors. Wu et al. showed that Tim-3 expression on CD4+ T cells and CD8+ T cells was elevated in ovarian cancer [3]. Han et al. reported that the level of Tim-3 on CD4+ T cells was increased in glioma patients and was correlated with disease progression [4]. It was reported that the co-expression of Tim-3 and PD-1 can be found in the tumor infiltrating lymphocytes of the solid tumor in mice [5]. These studies continue to demonstrate a strong correlation between Tim-3 expression and tumor-associated immune suppression. However, whether Tim-3 is also expressed on cancer cells, especially myeloid malignant cells, remains an open question. Accumulating studies have revealed the prognostic value of Tim-3 expression as well.

Tim-3 has been shown to be expressed in most leukemic stem cells (LSC) in the majority of non-M3 AML subtypes [6]. Tim-3+AML cells were also able to reconstitute AML and anti-human Tim-3 antibody blocked AML engraftment in axenotransplant model [7]. However, the role of Tim-3 expression on blast cells in AML has not been well understood. In this study, we investigated the Tim-3 expression on blasts of AML patients by flow cytometry. Our aim was to determine the role of Tim-3 expression in patients with de novo AML and the correlation with the response to the induction chemotherapy.

## RESULTS

### Tim-3 expression and association of its expression with clinical characteristics in AML

**Figure 1:**
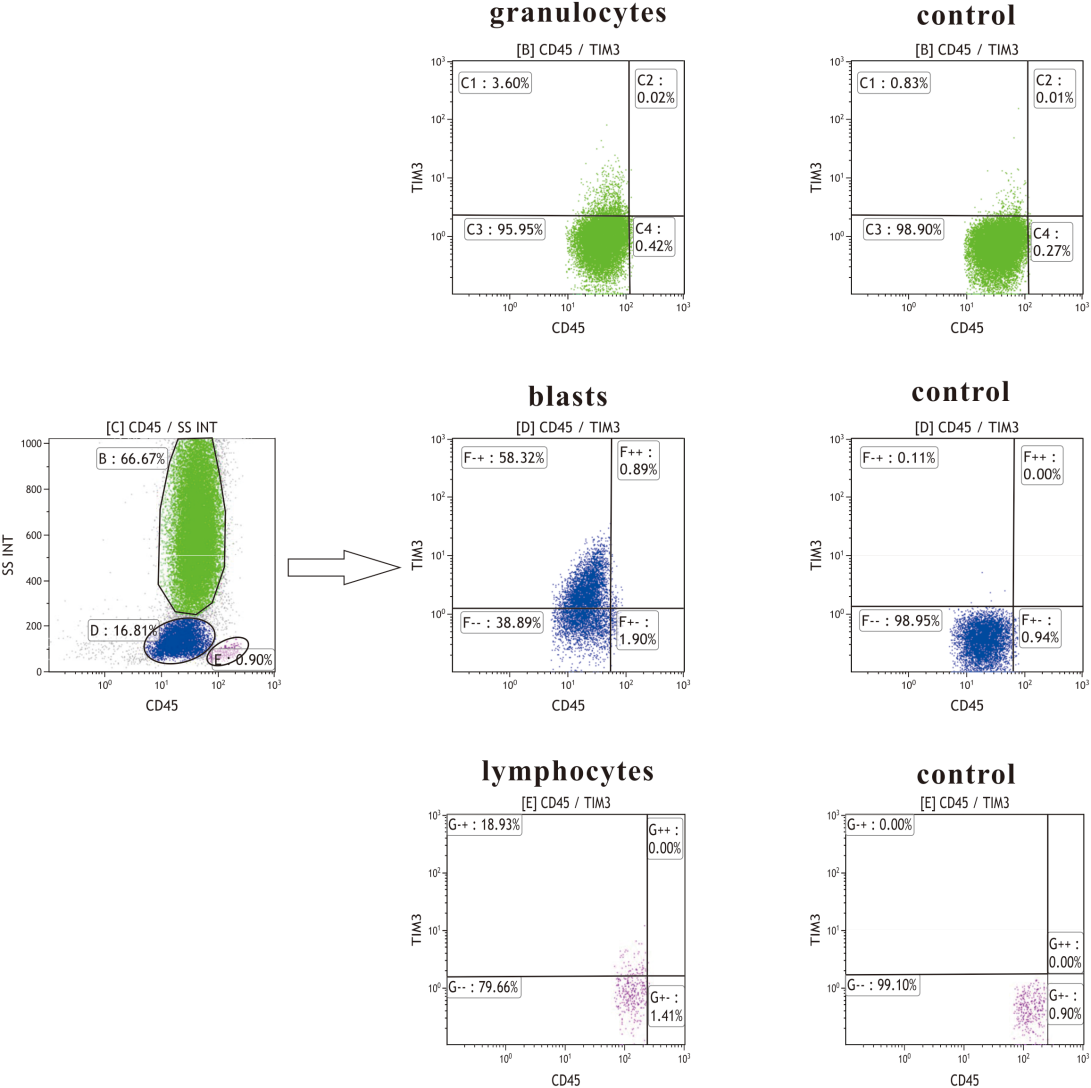
Tim-3 measurement strategy by flow cytometry

Tim-3 expression on blast cells was detected by flow cytometry (Figure 1). According to FAB type, 76 AML patients were diagnosed as M0 (n=2), M1 (n=16), M2 (n=20), M4 (n=20), M5 (n=16), and M6 (n=2), respectively. A positive expression (>20%) of Tim-3 was found in 87% (66/76) of patients with AML. The average percentage of Tim-3(+) blasts in these AML patients was 58.26 ± 29.23%. In order to evaluate the impact of Tim-3 expression levels on clinical outcome, AML patients were divided into low and high expresser groups based on their median Tim-3 expression levels. There was no significant difference in sex, age, hemoglobin concentration, WBC and platelet count, or percentage of blasts in bone marrow between these two groups. The frequency of Tim-3 high expression was higher in M4 patients than that in other AML patients according to FAB type (80% versus 39%, P=0.004). Besides, Tim-3 increased much more significantly in inv(16) than in other cytogenetic subgroups (100% versus 45%, P=0.01). In patients with high Tim-3 expression, frequency of C/EBPA mutation was significantly higher compared to that in low expressers (28% versus 8%, P=0.03). High levels of Tim-3 were found more frequently in low risk groups (76%) than in intermediate and high risk groups (38% and 41%, respectively) (P=0.02). But there was no significant difference in the mutations of the six other genes between low expressers and high expressers (Table 1). It was found that the expression of Tim-3 was also positively correlated with CD13 (r=0.36, P=0.001), CD34 (r=0.41, P=0.000) and CD7 (r=0.27, P=0.02).

**Table 1:** Tim-3 expression and clinical parameters in AML patients

Patient's parameters	Tim-3 expression status		*P*
	low (n=38)	high (n=38)	
Sex, male/female	18/20	19/19	0.89
Median age, years (range)	50(21-90)	52(15-80)	0.73
Median WBC, ×109/L (range)	21.5(0.8-308.4)	18.8(0.8-210.3)	0.62
Median hemoglobin, g/L (range)	99(48-163)	80(51-156)	0.17
Median platelets, ×109/L (range)	70(6-304)	66(9-202)	0.47
BM blasts, %(range)	63.5(21.0-96.0)	62.4(37.0-96.0)	0.91
FAB			0.03^*^
M0	2(5%)	0(0%)	0.16
M1	11(29%)	5(13%)	0.09
M2	12(32%)	8(21%)	0.30
M4	4(11%)	16(33%)	0.004^*^
M5	8(21%)	8(21%)	1.00
M6	1(3%)	1(3%)	1.00
Karyotype			0.12
Normal	22(58%)	16(42%)	0.20
T(8;21)	4(11%)	5(13%)	0.69
Inv(16)	0(0%)	6(16%)	0.01^*^
+8	2(5%)	1(3%)	0.57
Complex	4(11%)	3(8%)	0.72
Others	5(13%)	5(13%)	0.96
No data	1(3%)	2(5%)	
Gene Mutation			
C/EBPA(+/−)	3/33	10/26	0.03^*^
NPM1(+/−)	4/32	7/29	0.33
c-KIT(+/−)	2/34	1/35	0.56
FLT3-ITD (+/−)	7/29	5/31	0.53
IDH1/2(+/−)	2/34	3/33	0.65
DNMT3A(+/−)	3/33	2/34	0.65
ELN risk group			0.02^*^
Low risk group	5(13%)	16(42%)	0.004^*^
Intermediate risk group	18(53%)	11(29%)	0.09
High risk group	13(29%)	9(24%)	0.31
No data	2(5%)	2(5%)	

WBC, white blood cells; FAB, French-American-British classification; BM, bone marrow; ELN, European LeukemiaNet; ^*^, significantly statistically different.

### Response to induction chemotherapy and follow-up samples with CR

**Figure 2:**
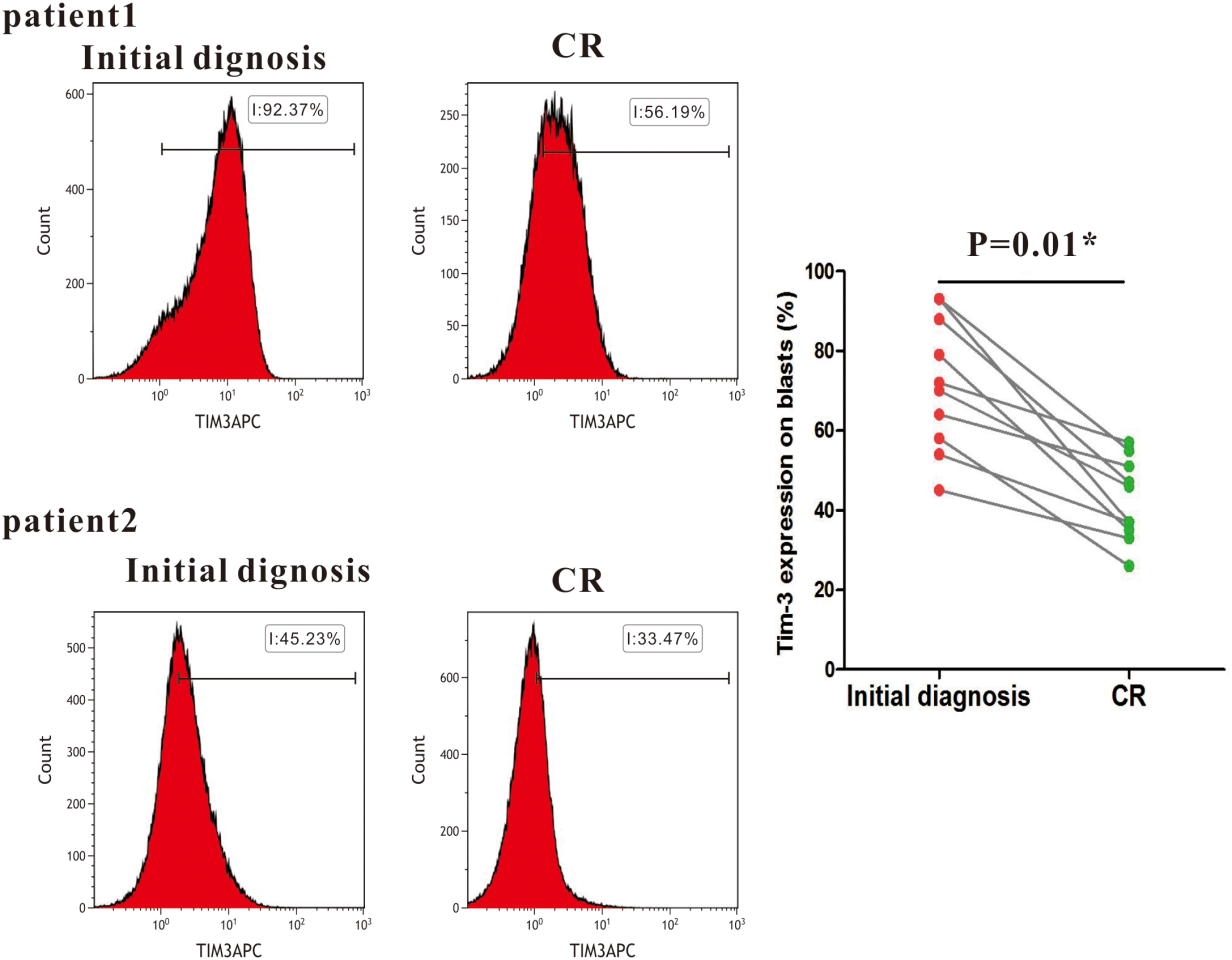
Changes of Tim-3 expression in 10 AML patients before and after CR

AML patients with high levels of Tim-3 expression achieved a higher rate of complete remission (CR) than patients with low levels of the expression (91% versus 67%, P=0.01). Ten samples of AML were monitored after CR. It was shown that Tim-3 expression significantly decreased after CR compared to that in the initial diagnosed AML patients (P=0.01) (Figure 2), suggesting that high levels of Tim-3 expression on blasts in AML may enhance the chemotherapy sensitivity of AML patients.

### Blockage of Tim-3 expression reduced cell apoptosis induced by chemotherapy

**Figure 3:**
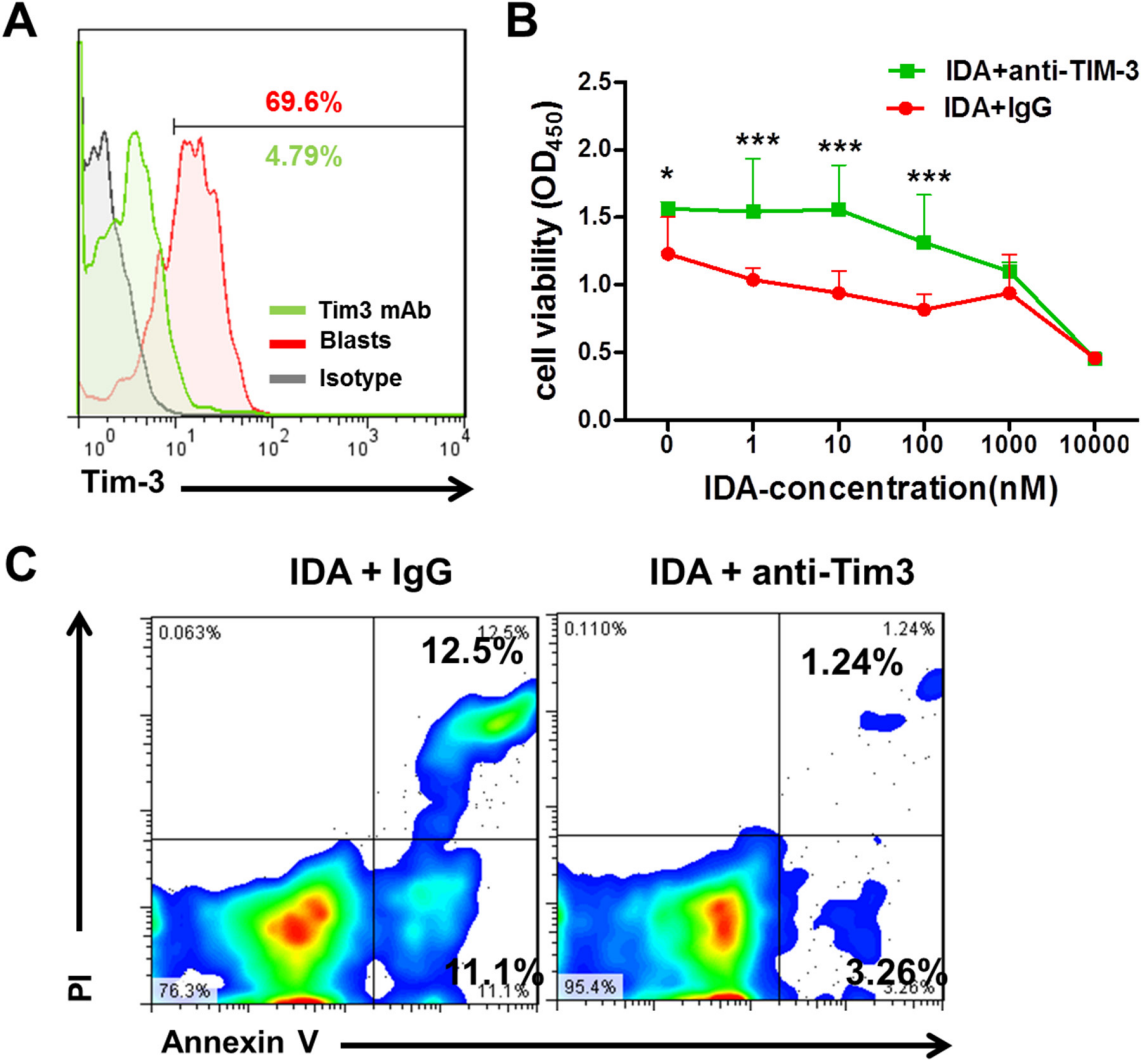
Effect of Tim-3 blockade on the proliferation and apoptosis of leukemic cells treated with IDA **(A)** Tim-3 expression was blocked efficiently in leukemic cells cultured from one AML patient. **(B)** Cell viability of leukemic cells at 48h was evaluated by CCK-8 assay with the anti-Tim-3 monoclonal antibody and IDA combined treatment. **(C)** Apoptosis of leukemic cells were determined by Annexin V/PI assays 48h after Tim-3 blockade and IDA combined treatment. ^*^, P<0.05, ^***^, P<0.001.

To further understand whether the expression levels of Tim-3 is relevant to chemotherapy sensitivity, we blockaded the expression of Tim-3 on AML blasts by mouse anti-human Tim-3 monoclonal antibody and then assessed the chemotherapy sensitivity. For this purpose, blasts with high levels of Tim-3 expression were isolated from the bone marrow of AML patients and treated with IDA in the presence of anti-Tim-3 antibody or control IgG (Figure 3A). The IDA treatment significantly decreased cell growth in dose-dependent effects; blockade of Tim-3 expression on AML blasts significantly reduced the IDA-mediated suppression of cell growth (Figure 3B). To determine whether the protective effects of Tim-3 blockade was associated with reduction of apoptosis in AML cells, we carried out Annexin V/PI apoptosis assays. The results showed that the percentages of apoptotic cells significantly decreased after the Tim-3 blockade compared to cells treated with IgG control (Figure 3C). These results suggest that Tim-3 may enhance the leukemic cells sensitization to the chemotherapy by IDA.

## DISCUSSION

T-cell immunoglobulin and mucin domain-containing molecule 3 (Tim-3) is a membrane protein expressed in various kinds of immune cells and plays a pivotal role in immune regulation [1, 13–15]. Recently, some reports about solid tumors showed that Tim-3 may be a potential prognostic biomarker and a novel therapeutic target since its expression was related to several clinicopathological parameters and prognosis. In bladder urothelial carcinoma, Tim-3 expression was reported to significantly correlate with advanced pathological grade and T stage [16]. Overexpression of Tim-3 in colon cancer, non-small cell lung cancer and cervical cancer are often associated with poor clinical outcome and poor survival [17–19].

In our study, we found that Tim-3 was highly expressed on blast cells in non-M3 AML. Interestingly, the frequency of Tim-3 expression was higher in M4 patients than in other AML patients according to FAB type. Meanwhile, our results showed that there was a significant correlation of Tim-3 with the progenitor marker CD34, the early myeloid marker CD13, and T-cell marker CD7 in AML.

When it comes to the value of Tim-3 in prognosis, we tried to investigate the relationship between Tim-3 and other prognostic factors first. Mutations in core binding transcription factors that regulate myeloid differentiation may directly regulate Tim-3 transcription or arrest leukemic cells in a stage of differentiation associated with high Tim-3 expression [20, 21]. Jan et al. also suggested that Tim-3 was more highly expressed in AML associated with core binding factor translocations, t (8;21) (q22;q22) and inv (16) or mutations in C/EBPA in AML cases [6]. Our results revealed that Tim-3 expression was significantly associated with inv (16) and C/EBPA mutation, whereas no obvious association between Tim-3 expression and t (8;21) was discovered. Although more cases should be analyzed to further confirm the correlation between Tim-3 and t (8;21), the findings mentioned above generally suggested that Tim-3 was likely to be a good prognosis marker in AML.

Furthermore, our results showed for the first time that high levels of Tim-3 expression were more frequent in low-risk groups than in other groups among AML samples. In addition, AML patients with high Tim-3 expression achieved higher rate of CR than those with low expression and the level of Tim-3 expression significantly decreased in patients who achieved CR after chemotherapy. All these findings support our observation that high Tim-3 expression may indicate good prognosis in AML.

To explain the above phenomena, we designed an experiment *in vitro* to evaluate the effect of Tim-3 blockade on the function of cell proliferation and apoptosis. Leukemic cells from five patients of AML were cultured for the experiment and the anti-Tim-3 monoclonal antibody was used to block Tim-3 expression on them. All these cells also received IDA treatment. Our findings showed that the OD value of all the leukemic cells were increased after Tim-3 blockade, whereas the apoptosis rate of cells after Tim-3 blockade were decreased compared to those unblocked in CCK-8 test. However, the role of Tim-3 in leukemogenesis still remains unknown. Anderson et al found that in myeloid cells (unlike in T-cells), ligand-dependent Tim-3 activation triggers TNF-α generation and secretion [15]. Moreover, Tim-3 may also induce the growth factor-like responses in human myeloid leukemia cells [22, 23]. The activation of TNF-α and growth factor-like responses may enhance pro-inflammatory signaling, and this may become more obvious in AML chemotherapy.

According to the above findings, our data suggest that Tim-3 is a marker for good prognosis in AML patients. However, Noureldien et al. found that Tim-3 might serve as a biomarker to predict AML aggressiveness [24]. Although their results were inconsistent with ours, some limitations of their study were overcome by ours. First, the scale of our study was larger than theirs (76 vs 40). Moreover, flow cytometry was applied to detect Tim-3 expression on blasts in our study because Tim-3 is also expressed on T cells while they used quantitative RT-PCR to detect Tim-3 expression on bone marrow mononuclear cells in AML patients. Finally, cytogenetic and molecular profiles of patients in our study were more complete than theirs.

In conclusion, Tim-3 could be potentially used in AML as a biomarker for disease monitoring and Tim-3 high expression may lead to a good response to induction chemotherapy in de novo AML. Further follow-up data, including survival analysis, need to be investigated to explore the prognostic impact of Tim-3 in AML patients.

## MATERIALS AND METHODS

### Patients

A total of 76 AML cases (M1 to M6 except for M3) were newly diagnosed by bone marrow MICM (morphology, immunology, cytogenetics, and molecular biology) tests and then treated between February 2015 and October 2015 at the First Affiliated Hospital of Soochow University. The demographic and clinical data of these patients were shown in Table 1. The diagnosis and classification of the patients were based on the revised French-American-British (FAB) classification and the 2008 World Health Organization (WHO) criteria [8, 9]. According to the 2017 ELN classifications based on cytogenetic and molecular characteristics, all AML patients were divided into low-, intermediate- and high-risk groups [10]. Immunological criteria for lineage affiliation and subtype were applied according to the ‘EGIL recommendations’ [11]. Genomic DNA was extracted from BM-derived mononuclear cells using the PurelinkTM Genomic DNA mini kit (Invitrogen, Carlsbad, CA, USA) according to the manufacturer's instructions. Gene mutations (including the c-kit exons 8 and 17, NPM1 exon 12, DNMT exons 2 and 3, FLT3 exons 14,15 and 20, NRAS exons 2 and 3, KRAS exons 2,3 and 4, IDH1/2 exon 4, C/EBPA) were directly sequenced from two directions by a 3730 DNA analyzer (Applied biosystems, Foster, CA, USA). The gene mutations were detected by PCR amplification of the entire or a portion of the coding region. 76 AML patients received IA regimen as the standard induction chemotherapy (3+7 regimens idarubicin 10mg/m^2^/day for three days, cytarabine 100mg/m^2^/day for seven days). We evaluated the curative effect after two cycles of standard induction chemotherapy. Complete remission (CR) was defined as bone marrow blasts< 5%, the absence of blasts with Auer rods, the absence of extramedullay disease, absolute neutrophil count >1.0×10^9^/L, platelet count >100×10^9^/L, and independence of red cell transfusions. Written informed consent was obtained from all patients. The study protocol was approved by the institutional review board of The First Affiliated Hospital of Soochow University Ethics Committee. All patients gave written informed consent to their participation in the study, which followed the ethical guidelines of the Declaration of Helsinki.

### Sample preparation and antibodies for flow cytometry

The number of total bone marrow cells was quantified by microscopy and adjusted to 2×10^6^ in each tube. The immunophenotypic analysis was performed on lysed whole bone marrow samples with conjugated mAbs. The following mAbs were used in the study: FITC-conjugated anti-CD7, anti-CD10, anti-CD14, anti-CD2; PE-conjugated anti-CD34, anti-CD20, anti-CD13, anti-CD117; PC5-conjugated anti-HLA-DR, anti-CD19, anti-CD33, anti-CD15; PC7-conjugated anti-CD45 (Beckman Coulter, California, USA), APC-conjugated anti-Tim-3 (R&D systems, Minnesota, USA). The flow cytometry staining strategy was performed as follows: CD7/CD34/HLA-DR/CD45, CD10/CD20/CD19/CD45, CD14/CD13/CD33/CD45, CD2/CD117/CD15/CD45. Isotype-matched nonreactive mouse mAbs at the same protein concentrations were used as negative controls in all experiments. The positively threshold was 20% for all markers except for cytoplasmic or intranuclear antigens, for which a 10% threshold was used. Data acquisition and analysis were performed on NAVIOS (Beckman Coulter, California, USA).

### Cytogenetic analysis

Bone marrow samples were processed using the standard 24-hr unstimulated cultures. A conventional R-banding assay was used for karyotypic analysis. 20 or more cells were analyzed to exclude clonal abnormalities, which were defined in accordance with the International System for Human Cytogenetic Nomenclature (ISCN 2005) guidelines [12].

### Tim-3 blockade and cell viability assay

Bone marrow samples of five patients of AML with high Tim-3 expression were collected in tubes containing preservative-free heparin. Leukemic cells were obtained by Ficoll-Histopaque density gradient centrifugation. 3 mL of Histopaque 1.077g/mL were placed into a 10 mL plastic centrifuge tube, overlaid with 3 mL anticoagulated blood diluted 1:1 with phosphate-buffered saline (PBS), and centrifuged at 400×g for 30 min at room temperature. Interphase mononuclear cells banded at the interface between the plasma and the histopaque were recovered, washed twice with PBS, and then resuspended in RPMI 1640 medium (Sigma) containing 20% heat-inactivated bovine serum.

Cells were counted and seeded in triplicate in 200 μl of medium in a 96-well plate at 2×10^4^ cells per well. Cells were treated with idarubicin (IDA) (1, 10, 100, 500, 1000 nM) in the presence of mouse anti-human Tim-3 monoclonal antibody (clone 2E2, BioLegend, San Diego, CA, USA) or immunoglobulin G (IgG) isotype control at a concentration of 10 μg/ml. Cell proliferation was determined by a Cell Counting Kit-8 assay (Dojindo, Japan) 48h after being seeded as instructed by the manufacturer. The absorbance was measured at 450 nm in a microplate reader (Bio-Tek, Winooski, VT, USA). Cell apoptosis was evaluated using Annexin V-FITC/PI detection kit KeyGen Biotech Co., Nanjing, China) according to the manufacturer's instructions. Data were acquired on a FACS Calibur flow cytometer (BD Biosciences, San Jose, CA, USA) and analyzed using Cell Quest software (BD Biosciences, San Jose, CA, USA).

### Statistical analysis

All statistical calculations were performed with SPSS software (SPSS 20.0, Chicago, IL). Data were expressed as¯x±s and t-test was introduced in the comparison of normality data. At the same time, Nonparametric test (Mann-Whitney U test) was applied to analyze non-normality data. During the comparison process of categorical variables, Pearson Chi-square analysis or Fisher exact test was employed. Moreover, the spearman correlation test was conducted to analyze correlation between Tim-3 expression and immunophenotypes. The significance of results was defined as a level of P<0.05 at both tails.
